# The Efficacy and Development of Students' Problem-Solving Strategies During Compulsory Schooling: Logfile Analyses

**DOI:** 10.3389/fpsyg.2018.00302

**Published:** 2018-03-09

**Authors:** Gyöngyvér Molnár, Benő Csapó

**Affiliations:** ^1^Department of Learning and Instruction, University of Szeged, Szeged, Hungary; ^2^MTA-SZTE Research Group on the Development of Competencies, University of Szeged, Szeged, Hungary

**Keywords:** complex problem solving, logfile analyses, exploration strategies, VOTAT strategies, latent class profiles

## Abstract

The purpose of this study was to examine the role of exploration strategies students used in the first phase of problem solving. The sample for the study was drawn from 3^rd^- to 12^th^-grade students (aged 9–18) in Hungarian schools (*n* = 4,371). Problems designed in the MicroDYN approach with different levels of complexity were administered to the students via the eDia online platform. Logfile analyses were performed to ascertain the impact of strategy use on the efficacy of problem solving. Students' exploration behavior was coded and clustered through Latent Class Analyses. Several theoretically effective strategies were identified, including the vary-one-thing-at-a-time (VOTAT) strategy and its sub-strategies. The results of the analyses indicate that the use of a theoretically effective strategy, which extract all information required to solve the problem, did not always lead to high performance. Conscious VOTAT strategy users proved to be the best problem solvers followed by non-conscious VOTAT strategy users and non-VOTAT strategy users. In the primary school sub-sample, six qualitatively different strategy class profiles were distinguished. The results shed new light on and provide a new interpretation of previous analyses of the processes involved in complex problem solving. They also highlight the importance of explicit enhancement of problem-solving skills and problem-solving strategies as a tool for knowledge acquisition in new contexts during and beyond school lessons.

## Introduction

Computer-based assessment has presented new challenges and opportunities in educational research. A large number of studies have highlighted the importance and advantages of technology-based assessment over traditional paper-based testing (Csapó et al., 2012). Three main factors support and motivate the use of technology in educational assessment: (1) the improved efficiency and greater measurement precision in the already established assessment domains (e.g., Csapó et al., 2014); (2) the possibility of measuring constructs that would be impossible to measure by other means (e.g., Complex Problem Solving (CPS)^1^; see Greiff et al., 2012, 2013); and (3) the opportunity of logging and analyzing not only observed variables, but metadata as well (Lotz et al., 2017; Tóth et al., 2017; Zoanetti and Griffin, 2017). Analyzing logfiles may contribute to a deeper and better understanding of the phenomenon under examination. Logfile analyses can provide answers to research questions which could not be answered with traditional assessment techniques.

This study focuses on problem solving, especially on complex problem solving (CPS), which reflects higher-order cognitive processes. Previous research identified three different ways to measure CPS competencies: (1) Microworlds (e.g., Gardner and Berry, 1995), (2) formal frameworks (Funke, 2001, 2010) and (3) minimal complex systems (Funke, 2014). In this paper, the focus is on the MicroDYN approach, which is a specific form of complex problem solving (CPS) in interactive situations using minimal complex systems (Funke, 2014). Recent analyses provide both a new theory and data-based evidence for a global understanding of different problem-solving strategies students employ or could employ in a complex problem-solving environment based on minimal complex systems.

The problem scenarios within the MicroDYN approach consist of a small number of variables and causal relations. From the perspective of the problem solver, solving a MicroDYN problem requires a sequence of continuous activities, in which the outcome of one activity is the input for the next. First, students interact with the simulated system, set values for the input variables, and observe the impacts of these settings on the target (dependent) variable. Then, they plot their conclusion about the causal relationships between the input and output variables on a graph (Phase 1). Next, they manipulate the independent variables again to set their values so that they result in the required values for the target variables (Phase 2).

When it comes to gathering information about a complex problem, as in the MicroDYN scenarios, there may be differences between the exploration strategies in terms of efficacy. Some of them may be more useful for generating knowledge about the system. Tschirgi (1980) identified different exploration strategies. When control of variables strategies (Greiff et al., 2014) were explored, findings showed that the vary-one-thing-at-a-time (VOTAT, Tschirgi, 1980; Funke, 2014) was the most effective strategy for identifying causal relations between the input and output variables in a minimal complex system (Fischer et al., 2012). Participants who employed this strategy tended to acquire more structural knowledge than those who used other strategies (Vollmeyer et al., 1996; Kröner et al., 2005). With the VOTAT strategy, the problem solver systematically varies only one input variable, while the others remain unchanged. This way, the effect of the variable that has just been changed can be observed directly by monitoring the changes in the output variables. There exist several types of VOTAT strategies.

Using this approach—defining the effectiveness of a strategy on a conceptual level, independently of empirical effectiveness—we developed a labeling system and a mathematical model based on all theoretically effective strategies. Thus, effectiveness was defined and linked to the amount of information extracted. An exploration strategy was defined as theoretically effective if the problem solver was able to extract all the information needed to solve the problem, independently of the application level of the information extracted and of the final achievement. We split the effectiveness of the exploration strategy and the usage and application of the information extracted to be able to solve the problem and control the system with respect to the target values based on the causal knowledge acquired. Systematicity was defined on the level of effectiveness based on the amount of information extracted and on the level of awareness based on the implementation of systematicity in time.

Students' actions were logged and coded according to our input behavior model and then clustered for comparison. We were able to distinguish three different VOTAT strategies and two successful non-VOTAT ones. We empirically tested awareness of the input behavior used in time. Awareness of strategy usage was analyzed by the sequence of the trials used, that is, by the systematicity of the trials used in time. We investigated the effectiveness of and differences in problem-solving behavior between three age groups by conducting latent class analyses to explore and define patterns in qualitatively different VOTAT strategy uses.

Although the assessment of problem solving within the MicroDYN approach is a relatively new area of research, its processes have already been studied in a number of different contexts, including a variety of educational settings with several age groups. Our cross-sectional design allows us to describe differences between age groups and outline the developmental tendencies of input behavior and strategy use among children in the age range covered by our data collection.

## Reasoning strategies in complex problem solving

Problem-solving skills have been among the most extensively studied transversal skills over the last decade; they have been investigated in the most prominent comprehensive international large-scale assessments today (e.g., OECD, 2014). The common aspects in the different theoretical models are that a problem is characterized by a gap between the current state and the goal state with no immediate solution available (Mayer and Wittrock, 1996).

Parallel to the definition of the so-called twenty first-century skills (Griffin et al., 2012), recent research on problem solving disregards content knowledge and domain-specific processes. The reason for this is that understanding the structure of unfamiliar problems is more effective when it relies on abstract representation schemas and metacognitive strategies than on specifically relevant example problems (Klahr et al., 2007). That is, the focus is more on assessing domain-general problem-solving strategies (Molnár et al., 2017), such as complex problem solving, which can be used to solve novel problems, even those arising in interactive situations (Molnár et al., 2013).

Logfile analyses make it possible to divide the continuum of a problem-solving process into several scoreable phases by extracting information from the logfile that documents students' problem-solving behavior. In our case, latent class analysis extracts information from the file that logs students' interaction with the simulated system at the beginning of the problem-solving process. The way students manipulate the input (independent) variables represents their reasoning strategy. Log data, on the one hand, make it possible to analyze qualitative differences in these strategies and then their efficiency in terms of how they generate knowledge resulting in the correct plotting of the causal relationship in Phase 1 and then the proper setting to reach the required target value in Phase 2. On the other hand, qualitative strategy data can be quantified, and an alternative scoring system can be devised.

From the perspective of the traditional psychometric approach and method of scoring, these problems form a test task consisting of two scoreable items. The first phase is a knowledge acquisition process, where scores are assigned based on how accurately the causal relationship was plotted. The second phase is knowledge application, where the correctness of the value for the target variable is scored. Such scoring based on two phases of solving MicroDYN problems has been used in a number of previous studies (e.g., Greiff et al., 2013, 2015; Wüstenberg et al., 2014; Csapó and Molnár, 2017; Greiff and Funke, 2017).

To sum up, there is great potential to investigate and cluster the problem-solving behavior and exploration strategy usage of the participants at the beginning of the problem-solving process and correlate the use of a successful exploration strategy with the model-building solution (achievement in Phase 1) observed directly in these simulated problem scenarios. Using logfile analyses (Greiff et al., 2015), the current article wishes to contribute insights into students' approaches to explore and solve problems related to minimal complex systems. By addressing research questions on the problem-solving strategies used, the study aims to understand students' exploration behavior in a complex problem-solving environment and the underlying causal relations. In this study, we show that such scoring can be developed through latent class analysis and that this alternative method of scoring may produce more reliable tests. Furthermore, such scoring can be automated and then employed in a large-scale assessment.

There are two major theoretical approaches to cognition relevant to our study; both offer general principles to interpret cognitive development beyond the narrower domain of problem solving. Piaget proposed the first comprehensive theory to explain the development of children's thinking as a sequence of four qualitatively different stages, the formal operational stage being the last one (Inhelder and Piaget, 1958), while the information processing approach describes human cognition by using terms and analogies borrowed from computer science. The information processing paradigm was not developed into an original developmental theory; it was rather aimed at reinterpreting and extending Piaget's theory (creating several Neo-Piagetian models) and synthesizing the main ideas of the two theoretical frameworks (Demetriou et al., 1993; Siegler, 1999). One of the focal points of these models is to explain the development of children's scientific reasoning, or, more closely, the way children understand how scientific experiments can be designed and how causal relationships can be explored by systematically changing the values of (independent) variables and observing their impact on other (target) variables.

From the perspective of the present study, the essential common element of cognitive developmental research is the control of variables strategy. Klahr and Dunbar (1988) distinguished two related skills in scientific thinking, hypothesis formation and experimental design, and they integrated these skills into a coherent model for a process of scientific discovery. The underlying assumption is that knowledge acquisition requires an iterative process involving both. System control as knowledge application tends to include both processes, especially when acquired knowledge turns out to be insufficient or dysfunctional (J. F. Beckmann, personal communication, August 16, 2017). Furthermore, they separated the processes of rule induction and problem solving, defining the latter as a search in a space of rules (Klahr and Dunbar, 1988, p. 5).

de Jong and van Joolingen (1998) provided an overview of studies in scientific discovery learning with computer simulations. They concluded that a number of specific skills are needed for successful discovery, like systematic variation of variable values, which is in a focus of the present paper, and the use of high-quality heuristics for experimentation. They identified several characteristic problems in the discovery process and stressed that learners often have trouble interpreting data.

In one of the earliest systematic studies of students' problem-solving strategies, Vollmeyer et al. (1996) explored the impact of strategy systematicity and effectiveness on complex problem-solving performance. Based on previous studies, they distinguished the VOTAT strategy from other possible strategies [Change All (CA) and Heterogeneous (HT) other strategies], as VOTAT allows systematic exploration of the behavior of a system and a disconfirmation of hypotheses. In one of their experiments, they examined the hypothesis that VOTAT was more effective for acquiring knowledge than less systematic strategies. According to the results, the 36 undergraduate students had clearly shown strategy development. After interacting with the simulated system in several rounds, they tended to use the VOTAT strategy more frequently. In a second experiment, it was also demonstrated that goal specificity influences strategy use as well (Vollmeyer et al., 1996).

Beckmann and Goode (2014) analyzed the systematicity in exploration behavior in a study involving 80 first-year psychology students and focusing on the semantic context of a problem and its effect on the problem solvers' behavior in complex and dynamic systems. According to the results, a semantically familiar problem context invited a high number of a priori assumptions on the interdependency of system variables. These assumptions were less likely tested during the knowledge acquisition phase, this proving to be the main barrier to the acquisition of new knowledge. Unsystematic exploration behavior tended to produce non-informative system states that complicated the extraction of knowledge. A lack of knowledge ultimately led to poor control competency.

Beckmann et al. (2017) confirmed research results by Beckmann and Goode (2014) and demonstrated how a differentiation between complexity and difficulty leads to a better understanding of the cognitive mechanism behind CPS. According to findings from a study with 240 university students, the performance differences observed in the context of the semantic effect were associated with differences in the systematicity of the exploration behavior, and the systematicity of the exploration behavior was reflected in a specific sequence of interventions. They argued that it is only the VOTAT strategy—supplemented with the vary-*none*-at-a-time strategy in the case of noting autonomous changes—that creates informative system state transitions which enable problem solvers to derive knowledge of the causal structure of a complex, dynamic system.

Schoppek and Fischer (2017) also investigated VOTAT and the related “PULSE” strategy (all input variables to zero), which enables the problem solver to observe the eigendynamics of the system in a transfer experiment. They proposed that besides VOTAT and PULSE, other comprehensive knowledge elements and strategies, which contribute to successful CPS, should be investigated.

In a study with 2^nd^- to 4^th^-grade students, Chen and Klahr found little spontaneous development when children interacted with physical objects (in situations similar to that of Piaget's experiments), while more direct teaching of the control of variables strategy resulted in good effect sizes and older children were able to transfer the knowledge they had acquired (improved control of variable strategy) to remote contexts (Chen and Klahr, 1999). In a more recent study, Kuhn et al. (2008) further extended the scope of studies on scientific thinking, identifying three further aspects beyond the control of variables strategy, including coordinating effects of multiple influences, understanding the epistemological foundations of science and engaging in argumentation. In their experiment with 91 6th-grade students, they explored how students were able to estimate the impact of five independent variables simultaneously on a particular phenomenon, and they found that most students considered only one or two variables as possible causes.

## Aims

In this paper, we explore several research questions on effective and less effective problem-solving strategies used in a complex problem-solving environment and detected by logfile analyses. We use logfile analyses to empirically test the success of different input behavior and strategy usage in CPS tasks within the MicroDYN framework. After constructing a mathematical model based on all theoretically effective strategies, which provide the problem solver with all the information needed to solve the problem, and defining several sub-strategies within the VOTAT strategy based on the amount of effort expended to extract the necessary information, we empirically distinguish different VOTAT and non-VOTAT strategies, which can result in good CPS performance and which go beyond the isolated variation strategy as an effective strategy for rule induction (Vollmeyer et al., 1996). We highlight the most and least effective VOTAT strategies used in solving MicroDYN problems and empirically investigate the awareness of the strategy used based on the sequence of the sub-strategies used. Based on these results, we conduct latent class analyses to explore and define patterns in qualitatively different VOTAT strategy uses.

We thus intend to answer five research questions:

RQ1: Does the use of a theoretically effective strategy occur prior to high performance? In other words, does the use of a theoretically effective strategy result in high performance?
RQ2: Do all VOTAT strategies result in a high CPS performance? What is the most effective VOTAT strategy?
RQ3: How does awareness of the exploration strategy used influence overall performance on CPS tasks?
RQ4: What profiles characterize the various problem solvers and explorers?
RQ5: Do exploration strategy profiles differ across grade levels, which represent different educational stages during compulsory schooling?

## Hypotheses

In this study, we investigated qualitatively different classes of students' exploration behavior in CPS environments. We used latent class analysis (LCA) to study effective and non-effective input behavior and strategy use, especially the principle of isolated variation, across several CPS tasks. We compared the effectiveness of students' exploration behavior based on the amount of information they extracted with their problem-solving achievement. We posed five separate hypotheses.

Hypothesis 1: We expect that high problem-solving achievement is not closely related to expert exploration behavior.

Vollmeyer et al. (1996) explored the impact of strategy effectiveness on problem-solving performance and reported that effectiveness correlated negatively and weakly to moderately with solution error (*r* = −0.32 and *r* = −0.54, *p* < 0.05). They reported that “most participants eventually adopted the most systematic strategy, VOTAT, and the more they used it, the better they tended to perform. However, even those using the VOTAT strategy generally did not solve the problem completely” (p. 88). Greiff et al. (2015) confirmed that different exploration behaviors are relevant to CPS and that the number of sub-strategies implemented was related to overall problem-solving achievement.

Hypothesis 2: We expect that students who use the isolated variation strategy in exploring CPS problems have a significantly better overall performance than those who use a theoretically effective, but different strategy.

Sonnleiter et al. (2017) noted that “A more effective exploration strategy leads to a higher system knowledge score and the higher the gathered knowledge, the better the ability to achieve the target values. Thus, system knowledge can be seen as a reliable and valid measure of students' mental problem representations” (p. 169). According to Wüstenberg et al. (2012), students who consistently apply the principle of isolated variation—the most systematic VOTAT strategy—in CPS environments show better overall CPS performance, compared to those who use different exploration strategies. Kröner et al. (2005) reported a positive correlation between using the principle of isolated variation and the likelihood of solving the overall problem.

Hypothesis 3: We expected that more aware CPS exploration behavior would be more effective than exploration behavior that generally results in extracting all the necessary information from the system to solve the problem, but within which the steps have no logically built structure and no systematicity in time.

Vollmeyer et al. (1996) explored the impact of strategy systematicity on problem-solving performance. They emphasized that “the systematicity of participants' spontaneous hypothesis-testing strategies predicted their success on learning the structure of the biology lab problem space” (p. 88). Vollmeyer and her colleagues restricted systematic strategy users to isolated variation strategy users; this corresponds to our terminology usage of aware isolated variation strategy users.

Hypothesis 4: We expected to find a distinct number of classes with statistically distinguishable profiles of CPS exploration behavior. Specifically, we expected to find classes of proficient, intermediate and low-performing explorers.

Several studies (Osman and Speekenbrink, 2011; Wüstenberg et al., 2012; Greiff et al., 2015) have indicated that there exist quantitative differences between different exploration strategies, which are relevant to a CPS environment. The current study is the first to investigate whether a relatively small number of qualitatively different profiles of students' exploration proficiency can be derived from their behavior detected in a CPS environment in a broad age range.

Hypothesis 5: We expected that more proficient CPS exploration behavior would be more dominant at later grade levels as an indication of cognitive maturation and of increasing abilities to explore CPS environments.

The cognitive development in children between Grades 3 and 12 is immense. According to Piaget's stage theory, they move from concrete operations to formal operations and they will be able to think logically and abstractly. According to Galotti (2011) and Molnár et al. (2013), the ability to solve problems effectively and to make decisions in CPS environments increases in this period of time; Grades 6–8 seem especially crucial for development. Thus, we expect that cognitive maturation will also be reflected in more proficient exploration behavior.

## Methods

### Participants

The sample was drawn from 3^rd^- to 12^th^-grade students (aged 9–18) in Hungarian primary and secondary schools (*N* = 4,371; Table 1). School classes formed the sampling unit. 180 classes from 50 schools in different regions were involved in the study, resulting in a wide-ranging distribution of students' background variables. The proportion of boys and girls was about the same.

**Table 1:** Composition of samples.

**Grade**	**Sample size**	**Gender, % female**	**Mean age (sd)**
3	584	–	–
4	679	–	–
5	608	–	–
6	677	49	11.92 (0.53)
7	607	51	12.94 (0.53)
8	942	49	13.89 (0.56)
9	30	48	15.00 (0.59)
10	84	51	16.79 (0.49)
11	102	68	17.02 (0.79)
12	58	64	17.93 (0.57)

### Materials

The MicroDYN approach was employed to develop a measurement device for CPS. CPS tasks within the MicroDYN approach are based on linear structural equations (Funke, 2001), in which up to three input variables and up to three output variables are related (Greiff et al., 2013). Because of the small set of input and output variables, the MicroDYN problems could be understood completely with precise causal analyses (Funke, 2014). The relations are not presented to the problem solver in the scenario. To explore these relations, the problem solver must interact directly with the problem situation by manipulating the input variables (Greiff and Funke, 2010), an action that can influence the output variables (direct effects), and they must use the feedback provided by the computer to acquire and employ new knowledge (Fischer et al., 2012). Output variables can change spontaneously and can consist of internal dynamics, meaning they can change without changing the input variables (indirect effects; Greiff et al., 2013). Both direct and indirect effects can be detected with an adequate problem-solving strategy (Greiff et al., 2012). The interactions between the problem situation and the test taker play an important role, but they can only be identified in a computerized environment based on log data collected during test administration.

In this study, different versions with different levels of item complexity were used (Greiff et al., 2013), which varied by school grade (Table 2; six MicroDYN scenarios were administered in total in Grades 3–4; eight in Grade 5: nine in Grades 6–8; and twelve in Grades 9–12); however, we only involved those six tasks where the principle of isolated variation was the optimal exploration strategy. That is, we excluded problems with an external manipulation-independent, internal dynamic effect or multiple dependence effect from the analyses, and there were no delayed or accumulating effects used in the problem environments created. Complexity was defined by the number of input and output variables and the number of relations based on Cognitive Load Theory (Sweller, 1994). “Findings show that increases in the number of relations that must be processed in parallel in reasoning tasks consistently lead to increases in task difficulty” (Beckmann and Goode, 2017).

**Table 2:** The design of the whole study: the complexity of the systems administered and the structure and anchoring of the tests applied in different grades.

**Complexity of the systems (number of input and output variables and connections without internal dynamics)**	**Presence of autoregressive dependencies**	**Grade**						
		**3**	**4**	**5**	**6**	**7**	**8**	**9–12**
2-1-2		+	+	+	+	+	+	+
2-2-2		+	+	+	+	+	+	+
2-2-2		+	+	+	+	+	+	+
2-2-2		+	+	+				
3-2-3		+	+	+	+	+	+	+
3-3-3		+	+	+	+	+	+	+
3-3-4								+
3-2-1	+			+	+	+	+	+
3-3-4				+	+	+	+	+
3-2-2	+				+	+	+	+
3-3-3	+				+	+	+	+
3-3-3	+							+
3-3-3	+							+

**Figure 1:**
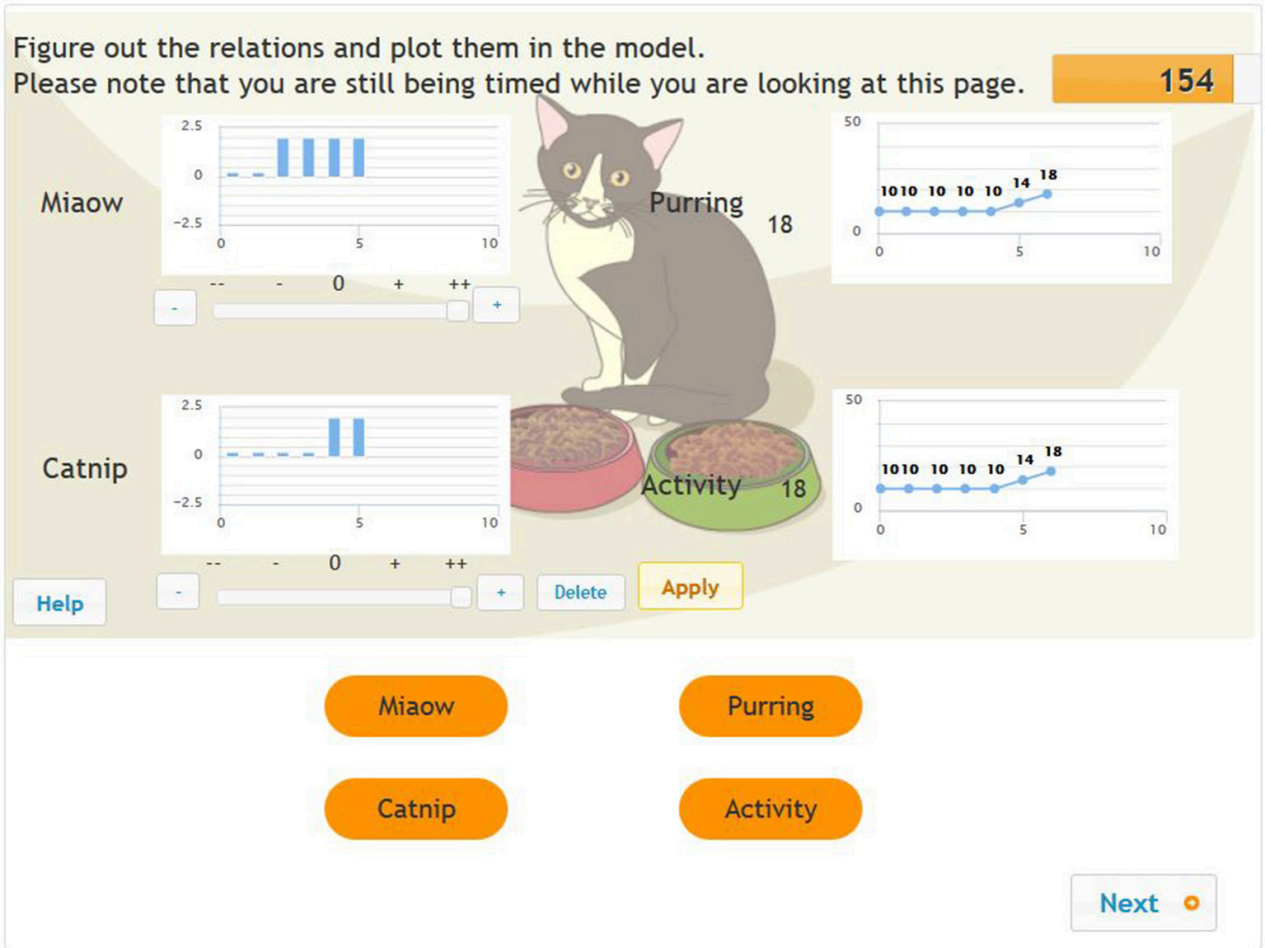
Exploration in phase 1 of the MicroDYN problems (two input variables and two output variables).

The tasks were designed so that all causal relations could be identified with systematic manipulation of the inputs. The tasks contained up to three input variables and up to three output variables with different fictitious cover stories. The values of the input variables were changed by clicking on a button with a + or – sign or by using a slider connected to the respective variable (see Figure 1). The controllers of the input variables range from “– –” (value = −2) to “++” (value = +2). The history of the values of the input variables within the same scenario was presented on a graph connected to each input variable. Beyond the input and output variables, each scenario contained a Help, Reset, Apply and Next button. The Reset button set the system back to its original status. The Apply button made it possible to test the effect of the currently set values of the input variables on the output variables, which appeared in the form of a diagram of each output variable. According to the user interface, within the same phase of each of the problem scenarios, the input values remained at the level at which they were set for the previous input until the Reset button was pressed or they were changed manually. The Next button implemented the navigation between the different MicroDYN scenarios and the different phases within a MicroDYN scenario.

In the knowledge acquisition phase, participants were freely able to change the values of the input variables and attempt as many trials for each MicroDYN scenario as they liked within 180 s. During this 180 s, they had to draw the concept map (or causal diagram; Beckmann et al., 2017); that is, they had to draw the arrows between the variables presented on the concept map under the MicroDYN scenario on screen. In the knowledge application phase, students had to check their respective system using the right concept map presented on screen by reaching the given target values within a given time frame (90 s) in no more than four trials, that is, with a maximum of four clicks on the Apply button. This applied equally to all participants.

### Procedures

All of the CPS problems were administered online via the eDia platform. At the beginning, participants were provided with instructions about the usage of the user interface, including a warm-up task. Subsequently, participants had to explore, describe and operate unfamiliar systems. The assessment took place in the schools' ICT labs using the available school infrastructure. The whole CPS test took approximately 45 min to complete. Testing sessions were supervised by teachers who had been thoroughly trained in test administration. Students' problem-solving performance in the knowledge acquisition and application phases was automatically scored as CPS performance indicators; thus, problem solvers received immediate performance feedback at the end of the testing session. We split the sample into three age groups, whose achievement differed significantly (Grades 3–5, *N* = 1,871; Grades 6–7, *N* = 1,284; Grades 8–12, *N* = 1,216; *F* = 122.56, *p* < 0.001; *t*_level_1_2_ = −6.22, *p* < 0.001; t_level_2_3_ = −8.92, *p* < 0.001). This grouping corresponds to the changes in the developmental curve relevant to complex problem solving. The most intensive development takes place in Grades 6–7 (see Molnár et al., 2013). Measurement invariance, that is, the issue of structural stability, has been demonstrated with regard to complex problem solving in the MicroDYN approach already (e.g., Greiff et al., 2013) and was confirmed in the present study (Table 3). Between group differences can be interpreted as true and not as psychometric differences in latent ability. The comparisons across grade levels are valid.

**Table 3:** Goodness of fit indices for measurement invariance of MicroDYN problems.

**Model**	**χ2**	**df**	**Δχ2**	**Δdf**	**p**	**CFI**	**TLI**	**RMSEA**
Configural invariance	119.71	42	–	–	–	0.980	0.987	0.039
Strong factorial invariance	126.33	45	7.37	3	>0.05	0.986	0.980	0.038
Strict factorial invariance	145.49	52	15.02	8	>0.05	0.980	0.976	0.042

*χ^2^ and df were estimated by the weighted least squares mean and variance adjusted estimator (WLSMV). Δχ^2^ and Δdf were estimated by the Difference Test procedure in MPlus. Chi-square differences between models cannot be compared by subtracting χ^2^s and dfs if WLSMV estimators are used. CFI, comparative fit index; TLI, Tucker Lewis index; RMSEA, root mean square error of approximation*.

The latent class analysis (Collins and Lanza, 2010) employed in this study seeks students whose problem-solving strategies show similar patterns. It is a probabilistic or model-based technique, which is a variant of the traditional cluster analysis (Tein et al., 2013). The indicator variables observed were re-coded strategy scores. Robust maximum likelihood estimation was used and two to seven cluster solutions were examined. The process of latent class analysis is similar to that of cluster analysis. Information theory methods, likelihood ratio statistical test methods and entropy-based criteria were used in reducing the number of latent classes. As a measure of the relative model fit, AIC (Akaike Information Criterion), which considers the number of model parameters, and BIC (Bayesian Information Criterion), which considers the number of parameters and the number of observations, are the two original and most commonly used information theory methods for model selection. The adjusted Bayesian Information Criterion (aBIC) is the sample size-adjusted BIC. Lower values indicated a better model fit for each criterion (see Dziak et al., 2012). Entropy represents the precision of the classification for individual cases. MPlus reports the relative entropy index of the model, which is a re-scaled version of entropy on a [0,1] scale. Values near one, indicating high certainty in classification, and values near zero, indicating low certainty, both point to a low level of homogeneity of the clusters. Finally, the Lo–Mendell–Rubin Adjusted Likelihood Ratio Test (Lo et al., 2001) was employed to compare the model containing n latent classes with that containing *n*−1 latent classes. A significant *p*-value (*p* < 0.05) indicates that the *n*−1 model is rejected in favor of a model with n classes, as it fits better than the previous one (Muthén and Muthén, 2012).

### Scoring

As previous research has found (Greiff et al., 2013), achievement in the first and second phases of the problem-solving process can be directly linked to the concept of knowledge acquisition (representation) and knowledge application (generating a solution) and was scored dichotomously. For knowledge acquisition, students' responses were scored as correct (“1”) if the connections between the variables were accurately indicated on the concept map (students' drawings fully matched the underlying problem structure); otherwise, the response was scored as incorrect (“0”). For knowledge application, students' responses were scored as correct (“1”) if students reached the given target values within a given time frame and in no more than four steps, that is, with a maximum of four clicks on the Apply button; otherwise, the response was scored as incorrect (“0”).

We developed a labeling procedure to divide the continuum of the problem-solving process into more scoreable phases and to score students' activity and behavior in the exploration phase at the beginning of the problem-solving process. For the different analyses and the most effective clustering, we applied a categorization, distinguishing students' use of the full, basic and minimal input behavior within a single CPS task (detailed description see later). The unit of this labeling process was a trial, a setting of the input variables, which was tested by clicking on the Apply button during the exploration phase of a problem, thus between receiving the problem and clicking on the Next button to reach the second part, the application part of the problem. The sum of these trials, within the same problem environment is called the input behavior. The input behavior was called a strategy if it followed meaningful regularities.

By our definition, the full input behavior model describes what exactly was done throughout the exploration phase and what kinds of trials were employed in the problem-solving process. It consists of all the activities with the sliders and Apply buttons in the order they were executed during the first phase, the exploration phase of the problem-solving process. The basic input behavior is part of the full input behavior model by definition, when the order of the trials attempted was still being taken into account, but it only consists of activities where students were able to acquire new information on the system. This means that the following activities and trials were not included in the basic input behavior model (they were deleted from the full input behavior model to obtain the basic behavior model):

- where the same scenario, the same slider adjustment, was employed earlier within the task (that is, we excluded the role of *ad hoc* control behavior from the analyses),
- where the value (position) of more than one input variable (slider) was changed and where the effect of the input variable on the operation of the system was still theoretically unknown to the problem solver,
- where a new setting or new slider adjustment was employed, though the effect of the input variables used was known from previous settings.
- As the basic input behavior involves timing, that is, the order of the trials used, it is suitable for the analyses with regard to the awareness of the input behavior employed.

Finally, we generated the students' minimal input behavior model from the full input behavior model. By our definition, the minimal input behavior focuses on those untimed activities (a simple list, without the real order of the trials), where students were able to obtain new information from the system and were able to do so by employing the most effective trials.

Each of the activities in which the students engaged and each of the trials which they used were labeled according to the following labeling system to be able to define students' full input behavior in a systematic format (please note that the numerical labels are neither scores nor ordinal or metric information):

- Only one single input variable was manipulated, whose relationship to the output variables was unknown (we considered a relationship unknown if its effect cannot be known from previous settings), while the other variables were set at a neutral value like zero. We labeled this trial +1.
- One single input variable was changed, whose relationship to the output variables was unknown. The others were not at zero, but at a setting used earlier. We labeled this trial +2.
- One single input variable was changed, whose relationship to the output variables was unknown, and the others were not at zero; however, the effect of the other input variable(s) was known from earlier settings. Even so, this combination was not attempted earlier. We labeled this trial +3.
- Everything was maintained in a neutral (zero) position. This trial is especially important for CPS problems with their own internal dynamics. We labeled this +A.
- The value of more than one input variable, whose relationship to the output variables was unknown, was changed at the same time, resulting in no additional information on the system. It was labeled –X.
- The same trial, the slider adjustment, had already been employed earlier within the task, resulting in no additional information on the system. It was labeled −0.
- A new slider adjustment was employed; however, the effect of the manipulated input variables was known from previous settings. This trial offered no additional information on the system and was labeled +0.

Although several input variables were changed by the scenario, it was theoretically possible to count the effect of the input variables on the output variables based on the information from the previous and present settings by using and solving linear equations. It was labeled +4.

An extra code (+5) was employed in the labeling process, but only for the basic input behavior, when the problem solver was able to figure out the structure of the problem based on the information obtained in the last trial used. This labeling has no meaning in the case of the minimal input behavior.

The full, basic and minimal input behavior models as well as the labeling procedure can be employed by analyzing problem solvers' exploration behavior and strategies for problems that are based on minimal complex systems. The user interface can preserve previous input values, and the values are not reset to zero after each exploration input. According to Fischer et al. (2012), VOTAT strategies are best for identifying causal relations between variables and they maximize the successful strategic behavior in minimal complex systems, such as CPS. By using a VOTAT strategy, the problem solver systematically varies only one input variable, while the others remain unchanged. This way, the effect of the changed variable can be found in the system by monitoring the changes in the output variables. There exist several types of VOTAT strategies based on the different combinations of VOTAT-centered trials +1, +2, and +3. The most obvious systematic strategy is when only one input variable is different from the neutral level in each trial and all the other input variables are systematically maintained at the neutral level. Thus, the strategy is a combination of so-called +1 trials, where it is employed for every input variable. Known as the isolated variation strategy (Müller et al., 2013), this strategy has been covered extensively in the literature. It must be noted that the isolated variation strategy is not appropriate to detect multiple dependence effects within the MicroDYN approach. We hypothesize that there are more and less successful input behaviors and strategies. We expect that theoretically effective, non-VOTAT strategies do not work as successfully as VOTAT strategies and that the most effective VOTAT strategy will be the isolated variation strategy.

We will illustrate the labeling and coding process and the course of generating a minimal input behavior out of a basic or full input behavior through the following two examples.

Figure 1 shows an example with two input variables and two output variables. (The word problem reads as follows: “When you get home in the evening, there is a cat lying on your doorstep. It is exhausted and can barely move. You decide to feed it, and a neighbor gives you two kinds of cat food, Miaow and Catnip. Figure out how Miaow and Catnip impact activity and purring.”). The student who mapped the operation of the system as demonstrated in the figure pressed the Apply button six times in all, using the various settings for the Miaow and Catnip input variables.

In mapping the system, the problem solver kept the value of both the input variables at 0 in the first two steps (making no changes to the base values of the input variables), as a result of which the values of the output variables remained unchanged. In steps 3 and 4, he set the value of the Miaow input variable at 2, while the value of the Catnip variable remained at 0 (the bar chart by the name of each variable shows the history of these settings). Even making this change had no effect on the values of the output variables; that is, the values in each graph by the purring and activity variables are constantly horizontal. In steps 5 and 6, the student left the value of the Miaow input variable at 2, but a value of 2 was added to this for the Catnip input variable. As a result, the values of both output variables (purring and activity) began to grow by the same amount. The coding containing all the information (the full input behavior) for this sequence of steps was as follows: +A, −0, +1, −0, +2, −0. The reason for this is since steps 2, 4, and 6 were repetitions of previous combinations, we coded them as −0. Step 3 involved the purest use of a VOTAT strategy [changing the value of one input variable at a time, while keeping the values of the other input values at a neutral level (+1)], while the trial used in step 5 was also a VOTAT strategy. After all, only the value of one input variable changed compared to step 4. This is therefore not the same trial as we described in step 3 (+2). After step 5, all the necessary information was available to the problem solver. The basic input behavior for the same sequence of steps was +A, +1, +2, since the rest of the steps did not lead the problem solver to acquire unknown information. Independently of the time factor, the minimal input behavior in this case was also +A, +1, +2. The test taker was able to access new information on the operation of the system through these steps. From the point of view of awareness, this +1+2 strategy falls under aware strategy usage, as the +1 and +2 sub-strategies were not applied far apart (excluding the simple repetition of the executed trials next to each other) from each other in time. A good indicator of aware strategy usage is if there is no difference between minimal and basic input behavior.

**Figure 2:**
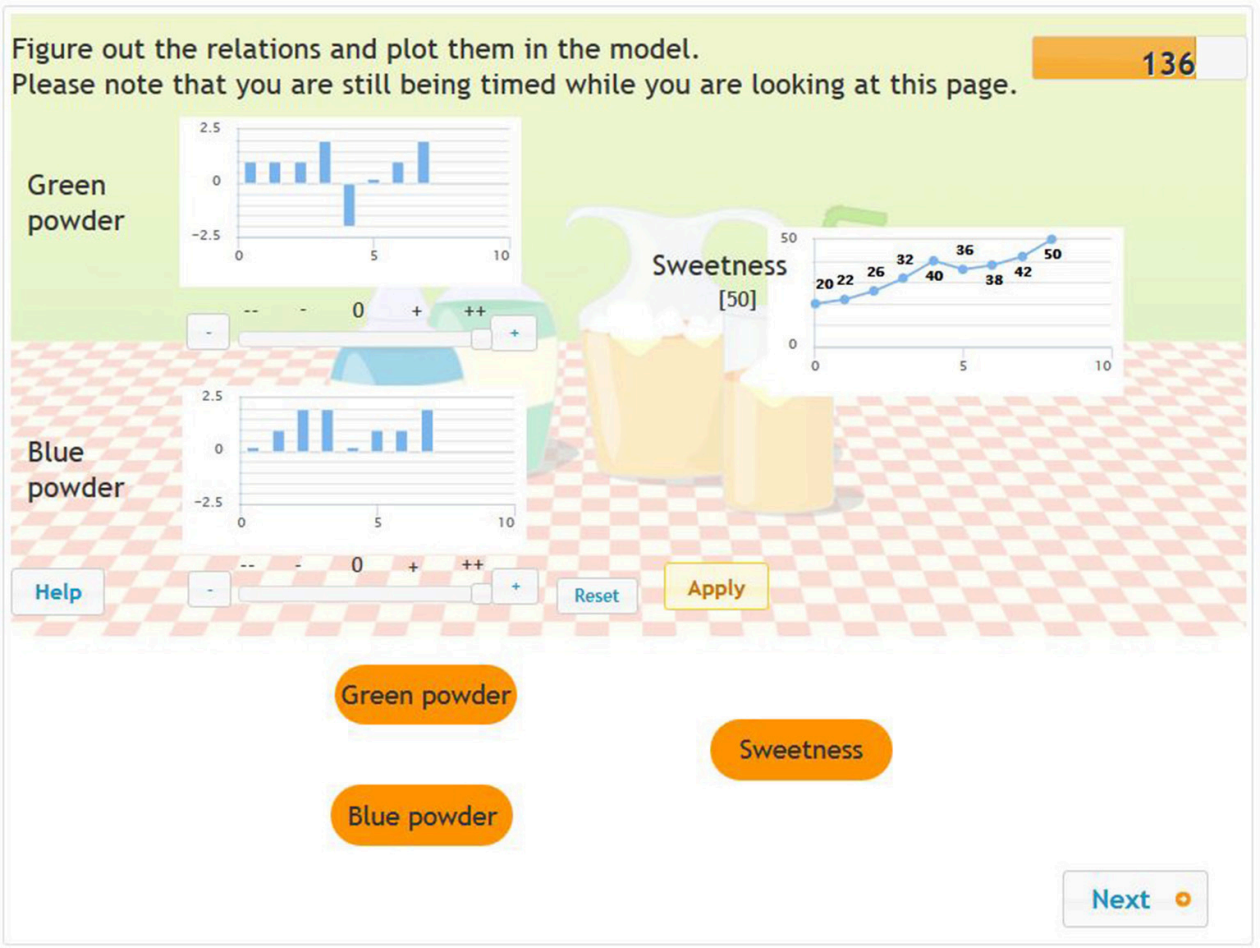
Exploration in phase 1 of the problems based on minimal complex systems (two input variables and one output variable).

In the second example (Figure 2), we demonstrate the sequence of steps taken in mapping another problem as well as the coding we used. Here the students needed to solve a problem consisting of two input variables and one output variable. The word problem reads as follows: “Your mother has bought two new kinds of fruit drink mix. You want to make yourself a fruit drink with them. Figure out how the green and blue powders impact the sweetness of the drink. Plot your assumptions in the model.” The test taker attempted eight different trials in solving this problem, which were coded as follows: +1, +2, +0, +0, +0, +0, −0, −0. After step 2, the student had access to practically all the information required to plot the causal diagram. (In step 1, the problem solver checked the impact of one scoop of green powder and left the quantity of blue powder at zero. Once mixed, the resultant fruit drink became sweeter. In step 2, the problem solver likewise measured out one scoop of green powder for the drink but also added a scoop of blue powder. The sweetness of the drink changed as much as it had in step 1. After that, the student measured out various quantities of blue and then green powder, and looked at the impact.) The basic input behavior coded from the full input behavior used by the problem solver was +1+2, and the minimal input behavior was +1+1 because the purest VOTAT strategy was used in steps 1 and 6. (Thus, both variables separately confirmed the effects of the blue and the green powder on the sweetness of the drink.) From the point of view of awareness, this +1+1 strategy falls under non-aware strategy usage, as the two applications of the +1 trial occurred far apart from each other in time.

Based on students' minimal input behavior we executed latent class analyses. We narrowed the focus to the principle of isolated variation, especially to the extent to which this special strategy was employed in the exploration phase as an indicator of students' ability to proficiently explore the problem environment. We added an extra variable to each of the problems, describing students' exploration behavior based on the following three categories: (1) no isolated variation at all (e.g., isolated variation was employed for none of the input variables – 0 points); (2) partially isolated variation (e.g., isolated variation was employed for some but not all the input variables – 1 point); and (3) fully isolated variation (e.g., isolated variation was employed for all the input variables – 2 points). Thus, depending on the level of optimal exploration strategy used, all the students received new categorical scores based on their input exploration behavior, one for each of the CPS tasks. Let us return to the example provided in Figures 1, 2. In the first example, a partially isolated strategy was applied, since the problem solver only used this strategy to test the effect of the Miaow input variables (in trials 3 and 4). In the second example, a full isolated strategy was applied, as the problem solver used this isolated variation strategy for both the input variables during the exploration phase in the first and sixth trials.

## Results

### The reliability of the test improved when scoring was based on the log data

The reliability of the MicroDYN problems as a measure of knowledge acquisition and knowledge application, the traditional CPS indicators for phases 1 and 2, were acceptable at α = 0.72–0.86 in all grades (Table 4). After we re-scored the problem solvers' behavior at the beginning of the problem-solving process, coded the log data and assigned new variables for the effectiveness of strategy usage during the exploration phase of the task for each task and person, the overall reliability of the test scores improved. This phenomenon was noted in all grades and in both coding procedures, when the amount of information obtained was examined (Cronbach's α ranged from 0.86 to 0.96) and when the level of optimal exploration strategy used was analyzed (Cronbach's α ranged from 0.83 to 0.98; the answers to the warm-up tasks were excluded from these analyses).

**Table 4:** Internal consistencies in scoring the MicroDYN problems: analyses based on both traditional CPS indicators and re-coded log data based on student behavior at the beginning of the problem-solving process.

**Grade**	**Reliabilities of the test by traditional scoring (phases 1 and 2)**	**Reliabilities of the test consisting of the new dichotomously scored variables in terms of the effectiveness of strategy usage at the beginning of the problem-solving process**	**Reliabilities of the test consisting of traditional scored items and the new dichotomously scored variables describing the effectiveness of strategy usage**	**Reliabilities of the test consisting of the new categorically scored variables describing the level of isolated variation strategy usage**
3	0.83	0.87	0.80	0.83
4	0.77	0.86	0.85	0.86
5	0.78	0.90	0.88	0.90
6	0.72	0.91	0.88	0.93
7	0.74	0.92	0.89	0.94
8	0.80	0.92	0.90	0.95
9	0.83	0.96	0.93	0.97
10	0.85	0.94	0.93	0.96
11	0.86	0.94	0.93	0.98
12	0.83	0.93	0.92	0.97

### Use of a theoretically effective strategy does not result in high performance (RQ1)

Use of a theoretically effective strategy did not always result in high performance. The percentage of effective strategy use and high CPS performance varied from 20 to 80%, depending on the complexity of the CPS tasks and the age group. The percentage of theoretically effective strategy use in each cohort increased by 20% for age when problems with the same complexity were compared (Table 5) and decreased about 20% for the increasing number of input variables in the problems.

**Table 5:** Percentage of theoretically effective and non-effective strategy use and high CPS performance.

**Complexity of problem (number of input and output variables and connections)**	**Frequency (%)**					
	**Theoretically effective strategy use**			**Theoretically non-effective strategy use**		
	**Low achievement (%; in proportion to whole sample)**	**High achievement (%; in proportion to whole sample)**	**Sum**	**Low achievement (%; in proportion to whole sample)**	**High achievement (%; in proportion to whole sample)**	**Sum**
**GRADES 3–5**						
2-1 (2)	19.9 (11.6)	80.1 (46.6)	58.2	28.2 (11.8)	71.8 (30.0)	41.8
2-2 (2)	81.5 (39.8)	18.5 (9.0)	50.2	97.2 (46.8)	2.8 (1.4)	49.8
3-2 (3)	65.9 (21.5)	34.1 (11.1)	32.6	89.3 (60.2)	10.7 (7.2)	67.4
3-3 (3)	60.2 (21.9)	39.8 (14.5)	36.4	77.1 (49.0)	22.9 (14.6)	63.6
**GRADES 6–7**						
2-1 (2)	28.3 (18.7)	71.6 (47.2)	65.9	26.9 (9.2)	73.1 (24.9)	34.1
2-2 (2)	72.4 (47.0)	27.5 (18.0)	59.0	98.2 (34.4)	1.8 (0.6)	41.0
3-2 (3)	50.8 (22.9)	49.2 (22.2)	45.0	85.9 (47.2)	14.1 (7.8)	54.9
3-3 (3)	52.6 (25.7)	47.4 (23.2)	49.0	77.3 (39.5)	22.7 (11.6)	51.0
**GRADES 8–12**						
2-1 (2)	28.7 (21.9)	71.3 (54.5)	76.4	25.5 (6.0)	74.5 (17.6)	23.6
2-2 (2)	59.4 (43.2)	40.6 (29.5)	72.7	98.2 (26.8)	1.8 (0.5)	27.3
3-2 (3)	42.0 (22.8)	58.0 (31.4)	54.2	81.9 (37.5)	18.1 (8.3)	45.8
3-3 (3)	39.4 (22.8)	60.6 (35.2)	58.0	74.1 (31.2)	25.8 (10.9)	42.0

The percentage of theoretically effective strategy use was the same for the less complex problems in Grades 3–5 and for the most complex tasks in Grades 8–12 (58%). More than 80% of these students solved the problem correctly in the first case, but only 60% had the correct solution in the second case. There was a 50% probability of effective and non-effective strategy use for problems with two input and two output variables in Grades 3–5 and for problems with three input and three output variables in Grades 6–7. In Grades 8–12, the use of a theoretically effective strategy was always higher than 50%, independently of the complexity of the problems (with no internal dynamic). The guessing factor, that is, the *ad hoc* optimization (use of a theoretically non-effective strategy with the correct solution) also changed, mostly based on the complexity and position of the tasks in the test. The results confirmed our hypothesis that the use of a theoretically effective strategy does not necessary represent the correct solution and that the correct solution does not always represent the use of an even theoretically effective problem-solving strategy.

### Not all the VOTAT strategies result in high CPS performance (RQ2)

**Figure 3:**
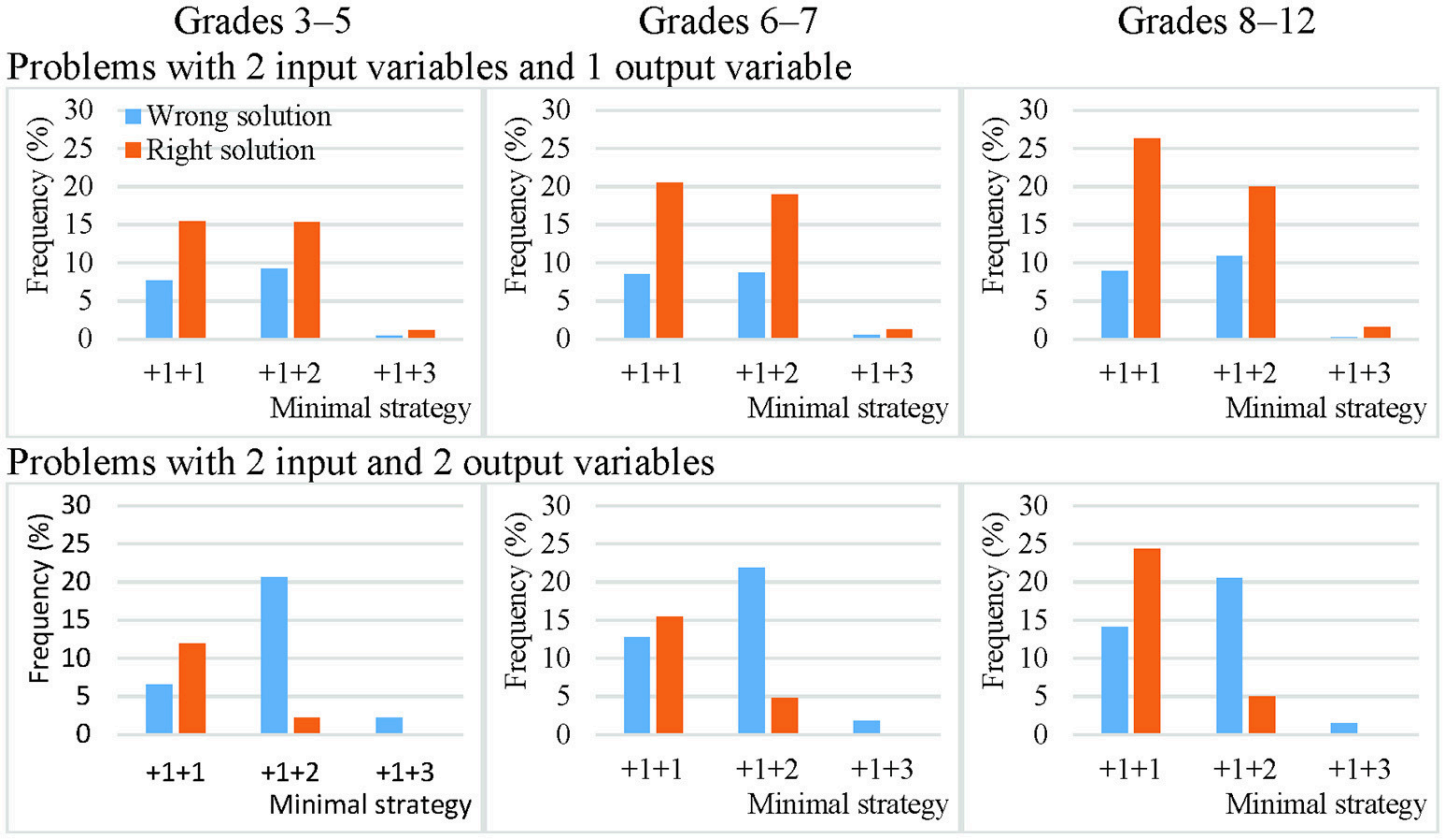
Efficacy of the most frequently employed VOTAT strategies on problems with two input variables and one or two output variables in Grades 3–5, 6–7, and 8–12.

**Figure 4:**
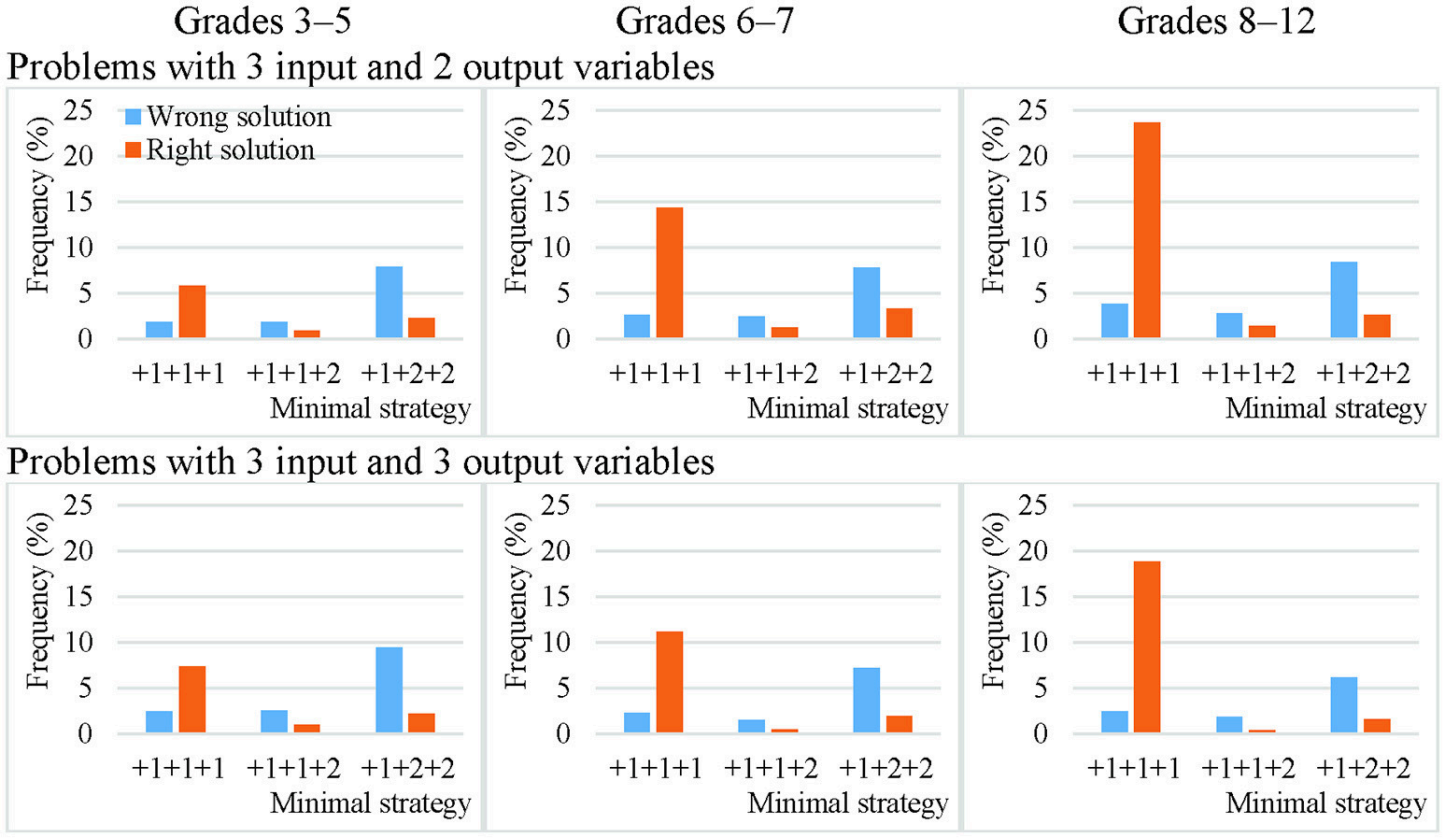
Efficacy of the most frequently employed VOTAT strategies on problems with three input variables and one or two output variables in Grades 3–5, 6–7, and 8–12.

On average, only 15% of the theoretically effective strategy uses involved non-VOTAT strategies. The isolated variation strategy comprised 45% of the VOTAT strategies employed. It was the only theoretically effective strategy which always resulted in the correct solution to the problem with higher probability independently of problem complexity or the grade of the students. The real advantage of this strategy was most remarkable in the case of the third cohort, where an average of 80% of the students who employed this strategy solved the problems correctly (Figures 3, 4).

The second most frequently employed and successful VOTAT strategy was the +1+2 type or the +1+2+2 type, depending on the number of input variables. In the +1+2 type, only one single input variable was manipulated in the first step, while the other variable remained at a neutral value; in the second step, only the other input variable was changed and the first retained the setting used previously. This proved to be relatively successful on problems with a low level of complexity independently of age, but it generally resulted in a good solution with a low level of probability on more complex problems.

VOTAT strategies of the +1+3 type (in the case of two input variables) and of the +1+1+2 type (in the case of three input variables) were employed even less frequently and with a lower level of efficacy than all the other VOTAT strategies (+1+1+3, +1+2+1, +1+2+2, +1+2+3, +1+3+1, +1+3+2 and +1+3+3 in the case of three input variables) and theoretically effective, non-VOTAT strategies (e.g., +4 in the case of two input variables or +1+4, +4+2 and +4+3 in the case of three input variables). In the following, we provide an example of the +4+2 type, where the MicroDyn problem has three input variables (A, B, and C) and three output variables. In the first trial, the problem solver set the input variables to the following values: 0 (for variable A), 1 (for variable B), and 1 (for variable C); that is, he or she changed two input variables at the same time. In the second trial, he or she changed the value of two input variables at the same time again and applied the following setting: 0 (for variable A), −2 (for variable B), and −1 (for variable C). In the third trial, he set variable A to 1, and left variables B and C unchanged. That is, the problem solver's input behavior can be described with the following trials: –X +4 +2. Based on this strategy, it was possible to map the relationships between the input and output variables without using any VOTAT strategy in the exploration phase.

### Aware explorers perform significantly higher on the CPS tasks (RQ3)

We compared the achievement of the aware, isolated strategy users with that of the non-aware explorers (Table 6). The percentage of high achievers among the non-aware explorers seemed to be almost independent of age, but strongly influenced by the complexity of the problem and the learning effect we noted in the testing procedure (see RQ5). Results for problems with two input variables and one output variable confirmed our previous results, which showed that the probability of providing the correct solution is very high even without aware use of a theoretically effective strategy (60–70%). With more complex problems, the difference between the percentages of aware and non-aware explorers was huge. Generally, 85% of the non-aware explorers failed on the problems, while at least 80% of the aware, isolated strategy users were able to solve the problems correctly.

**Table 6:** Percentage of high achievers among aware and non-aware explorers by grade and problem complexity.

**Complexity of problem**	**Grades 3–5**			**Grades 6–7**			**Grades 8–12**		
	**Percentage of high achievers among non- aware explorers**	**Percentage of high achievers among aware explorers using isolated variation strategy**	**Differences**	**Percentage of high achievers among aware explorers**	**Percentage of high achievers among aware explorers using isolated variation strategy**	**Differences**	**Percentage of high achievers among non- aware explorers**	**Percentage of high achievers among aware explorers using isolated variation strategy**	**Differences**
2-1	57.99	74.51	16.52	70.72	78.98	8.26	72.29	83.43	11.14
2-2	3.59	62.94	59.35	2.87	70.55	67.68	3.52	80.92	77.4
3-2	12.04	77.88	65.84	17.91	84.51	66.6	19.28	88.29	69.01
3-3	24.68	79.47	54.79	23.17	88.61	65.44	26.84	91.28	64.44

### Six qualitatively different explorer class profiles can be distinguished at the end of the elementary level and five at the end of the secondary level (RQ4 and RQ5)

In all three cohorts, each of the information theory criteria used (AIC, BIC, and aBIC) indicated a continuous decrease in an increasing number of latent classes. The likelihood ratio statistical test (Lo–Mendell Rubin Adjusted Likelihood Ratio Test) showed the best model fit in Grades 3–5 for the 4-class model, in Grades 6–7 for the 6-class model and in Grades 8–12 for the 5-class model. The entropy-based criterion reached the maximum values for the 2- and 3-class solutions, but it was also high for the best-fitting models based on the information theory and likelihood ratio criteria. Thus, the entropy index for the 4-class model showed that 80% of the 3^rd^- to 5^th^-graders, 82% of the 6^th^- to 7^th^-graders and 85% of the 8^th^- to 12^th^-graders were accurately categorized based on their class membership (Table 7).

**Table 7:** Information theory, likelihood ratio and entropy-based fit indices for latent class analyses.

**Number of latent classes**	**AIC**	**BIC**	**aBIC**	**Entropy**	**L–M–R test**	***p***
**GRADES 3–5**						
2	13,383	13,512	13,433	0.854	2,301	0.001
3	12,687	12,883	12,763	0.870	714	0.001
*4*	*12,563*	*12,826*	*12,664*	*0.800*	*149*	*0.006*
5	12,448	12,778	12,574	0.766	139	0.051
6	12,362	12,758	12,514	0.782	110	0.100
**GRADES 6–7**						
2	13,383	13,512	13,433	0.854	2,301	0.001
3	12,751	12,947	12,826	0.873	1,068	0.001
4	12,576	12,840	12,678	0.819	198	0.001
5	12,497	12,827	12,624	0.814	104	0.004
*6*	*12,427*	*12,824*	*12,580*	*0.823*	*95*	*0.022*
7	12,402	12,866	12,580	0.828	50	0.498
**GRADES 8–12**						
2	8,232	8,319	8,265	0.941	2,197	0.001
3	7,718	7,850	7,768	0.856	524	0.001
4	7,690	7,869	7,757	0.829	44	0.002
*5*	*7,686*	*7,911*	*7,771*	*0.853*	*21*	*0.003*
6	7,705	7,976	7,807	0.770	4	0.561

*AIC, Akaike Information Criterion; BIC, Bayesian Information Criterion; aBIC, adjusted Bayesian Information Criterion; L–M–R test, Lo–Mendell–Rubin Adjusted Likelihood Ratio Test. The best fitting model solution is in italics*.

We distinguished four latent classes in the lower grades based on the exploration strategy employed and the level of isolated variation strategy used (Table 8): 40.5% of the students proved to be non-performing explorers on the basis of their strategic patterns in the CPS environments. They did not use any isolated or partially isolated variation at all; 23.6% of the students were among the low-performing explorers who only rarely employed a fully or partially isolated variation strategy (with 0–20% probability on the less complex problems and 0–5% probability on the more complex problems). 24.7% of the 3^rd^- to 5^th^-graders were categorized as slow learners who were intermediate performers with regard to the efficiency of the exploration strategy they used on the easiest problems with a slow learning effect, but low-performing explorers on the complex ones. In addition, 11.1% of the students proved to be proficient explorers, who used the isolated or partially isolated variation strategy with 80–100% probability on all the proposed CPS problems.

**Table 8:** Relative frequencies and average latent class probabilities across grade levels 3–5, 6–7, and 8–12.

**Profiles**	**Grades 3–5**		**Grades 6–7**		**Grades 8–12**	
	**Frequency**	**Average latent class probabilities**	**Frequency**	**Average latent class probabilities**	**Frequency**	**Average latent class probabilities**
Non-performers	40.5	0.94	30.9	0.92	32.2	0.92
Low performers	23.6	0.86	14.0	0.84	16.2	0.87
Intermediate performers on easiest problems, but low performers on complex ones with a very slow learning effect	24.7	0.82	26.2	0.86	–	–
Rapid learners	–	–	4.4	0.86	7.7	0.96
Almost high performers on easiest problems, but low performers on complex ones with a slow learning effect	–	–	10.3	0.82	17.6	0.79
Proficient strategy users	11.1	0.97	14.2	0.96	26.3	0.97

In Grades 6–7, in which achievement proved to be significantly higher on average, 10% fewer students were observed in each of the first two classes (non-performing explorers and low-performing explorers). The percentage of intermediate explorers remained almost the same (26%), and we noted two more classes with the analyses: the class of rapid learners (4.4%) and that of slow learners, who are almost proficient explorers on the easiest problems, employing the fully or partially isolated variation strategy with 60–80% probability, but low-performing explorers on the complex ones (10.3%). The frequency of proficient strategy users was also increased (to 14.2%) compared to students in the lower grades. Finally, there was almost no change detected in the low-performing explorers' classes in Grades 8–12. We did not detect anyone in the class of intermediate explorers; they must have developed further and become (1) rapid learners (7.7%), (2) slow learners with almost high achievement with regard to the exploration strategy they used on the easiest problems, but low achievers on the complex ones (17.6%), or (3) proficient strategy users (26.3%), whose achievement was high both on the simplest and the most complex problems.

Based on these results, the percentage of non- and low explorers, who have very low exploration skills and do not learn during testing, decreased from almost 65 to 50% between the lower and higher primary school levels and then remained constant at the secondary level. There was a slight increase in respect of the percentage of students among the rapid learners. The students in that group used the fully or partially isolated strategy at very low levels at the beginning of the test, but they learned very quickly and detected these effective exploration strategies; thus, by the end of the test, their proficiency level with regard to exploration was equal to the top performers' achievement. However, we were unable to detect the class of rapid learners among 3^rd^- to 5^th^-graders.

Generally, students' level of exploration expertise with regard to fully and partially isolated variation improved significantly with age (*F* = 70.376, *p* < 0.001). According to our expectations based on the achievement differences among students in Grades 3–5, 6–8 and 9–12, there were also significant differences in the level of expertise in fully or partially isolated strategy use during problem exploration between 3^rd^- to 5^th^- and 6^th^- to 7^th^-grade students (*t* = −6.833, *p* < 0.001, *d* = 0.03) and between 6^th^- to 7^th^- and 8^th^- to 12^th^-grade students (*t* = −6.993, *p* < 0.001, *d* = 0.03).

## Discussion

In this study, we examined 3^rd^- to 12^th^-grade (aged 9–18) students' problem-solving behavior by means of a logfile analysis to identify qualitatively different exploration strategies. Students' activity in the first phase of the problem-solving process was coded according to a mathematical model that was developed based on strategy effectiveness and then clustered for comparison. Reliability analyses of students' strategy use indicated that strategies used in the knowledge acquisition phase described students' development (ability level) better than traditional quantitative psychometric indicators, including the goodness of the model. The high reliability indices indicate that there are untapped possibilities in analyzing log data. Our analyses of logfiles extracted from a simulation-based assessment of problem solving have expanded the scope of previous studies and made it possible to identify a central component of children's scientific reasoning: the way students understand how scientific experiments can be designed and how causal relationships can be explored by systematically changing the values of (independent) variables and observing their impact on other (target) variables.

In this way, we have introduced a new labeling and scoring method that can be employed in addition to the two scores that have already been used in previous studies. We have found that using this scoring method (based on student strategy use) improves the reliability of the test. Further studies are needed to examine the validity of the scale based on this method and to determine what this scale really measures. We may assume that the general idea of varying the values of the independent variables and connecting them to the resultant changes in the target variable is the essence of scientific reasoning and that the systematic manipulation of variables is related to combinatorial reasoning, while summarizing one's observations and plotting a model is linked to rule induction. Such further studies have to place CPS testing in the context of other cognitive tests and may contribute to efforts to determine the place of CPS in a system of cognitive abilities (see e.g., Wüstenberg et al., 2012).

We have found that the use of a theoretically effective strategy does not always result in high performance. This is not surprising, and it confirms research results by de Jong and van Joolingen (1998), who argue that learners often have trouble interpreting data. As we observed earlier, using a systematic strategy requires combinatorial thinking, while drawing a conclusion from one's observations requires rule induction (inductive reasoning). Students showing systematic strategies but failing to solve the problem may possess combinatorial skills but lack the necessary level of inductive reasoning. It is more difficult to find an explanation for the other direction of discrepancy, when students actually solve the problem without an effective (complete) strategy. Thus, solving the problem does not require the use of a strategy which provides the problem solver with sufficient information about the problem environment to be able to form the correct solution. This finding is similar to results from previous research (e.g., Vollmeyer et al., 1996; Greiff et al., 2015). Goode and Beckmann (2010) reported two qualitatively different, but equally effective approaches: knowledge- based and *ad hoc* control.

In the present study, the contents of the problems were not based on real knowledge, and the causal relationships between the variables were artificial. Content knowledge was therefore no help to the students in filling the gap between the insufficient information acquired from interaction and the successful solution to the problem. We may assume that students guessed intuitively in such a case. Further studies may ascertain how students guess in such situations.

The percentage of success is influenced by the complexity of the CPS tasks, the type of theoretically effective strategy used, the age group and, finally, the degree to which the strategy was consciously employed.

The most frequently employed effective strategies fell within the class of VOTAT strategies. Almost half the VOTAT strategies were of the isolated variation strategy type, which resulted with higher probability in the correct solution independently of the complexity of the problem or the grade of the students. As noted earlier, not all the VOTAT strategies resulted in high CPS performance; moreover, all the other VOTAT strategies proved to be significantly less successful. Some of them worked with relative success on problems with a low level of complexity, but failed with a high level of probability on more complex problems independently of age group. Generally, the advantage of the isolated variation strategy (Wüstenberg et al., 2014) compared to the other VOTAT and non-VOTAT, theoretically effective strategies is clearly evident from the outcome. The use of the isolated variation strategy, where students examined the effect of the input variables on the output variables independently, resulted in a good solution with the highest probability and proved to be the most effective VOTAT strategy independently of student age or problem complexity.

Besides the type of strategy used, awareness also played an influential role. Aware VOTAT strategy users proved to be the most successful explorers. They were followed in effectiveness by non-aware VOTAT strategy users and theoretically effective, but non-VOTAT strategy users. They managed to represent the information that they had obtained from the system more effectively and made good decisions in the problem-solving process compared to their peers.

We noted both qualitative and quantitative changes of problem-solving behavior in the age range under examination. Using latent class analyses, we identified six qualitatively different class profiles during compulsory schooling. (1) Non-performing and (2) low-performing students who usually employed no fully or partially isolated variation strategy at all or, if so, then rarely. They basically demonstrated unsystematic exploration behavior. (3) Proficient strategy users who consistently employed optimal exploration strategies from the very first problem as well as the isolated variation strategy and the partially isolated variation, but only seldom. They must have more elaborated schemas available. (4) Slow learners who are intermediate performers on the easiest problems, but low performers on the complex ones or (5) high performers on the easiest problems, but low performers on the complex ones. Most members of this group managed to employ the principle of isolated or partially isolated variation and had an understanding of it, but they were only able to use it on the easiest task and then showed a rapid decline on the more complex CPS problems. They might have been cognitively overloaded by the increasingly difficult problem-solving environments they faced. (6) Rapid learners, a very small group from an educational point of view. These students started out as non-performers in their exploration behavior on the first CPS tasks, showed a rapid learning curve afterwards and began to use the partially isolated variation strategy increasingly and then the fully isolated variation strategy. By the end of the test, they reached the same high level of exploration behavior as the proficient explorers. We observed no so-called intermediate strategy users, i.e., those who used the partially isolated variation strategy almost exclusively on the test. As we expected, class membership increased significantly in the more proficient classes at the higher grade levels due to the effects of cognitive maturation and schooling, but this did not change noticeably in the two lowest-level classes.

Limitations of the study include the low sample size for secondary school students; further, repetition is required for validation. The generalizability of the results is also limited by the effects of semantic embedding (i.e., cover stories and variable labels), that is, the usage of different fictitious cover stories “with the intention of minimizing the uncontrollable effects of prior knowledge, beliefs or suppositions” (Beckmann and Goode, 2017). An assumption triggered by semantic contexts has an impact on exploration behavior (e.g., the range of interventions, or strategies employed by the problem solver; Beckmann and Goode, 2014), that is, how the problem solver interacts with the system. Limitations also include the characteristics of the interface used. In our view, analyses with regard to VOTAT strategies are only meaningful in systems with an interface where inputs do not automatically reset to zero from one input to the next (Beckmann and Goode, 2017). That is, we excluded problem environments from the study where the inputs automatically reset to zero from one input to the next. A further limitation of the generalizability of the results is that we have omitted problems with autonomic changes from the analyses.

The main reason why we have excluded systems that contain autoregressive dependencies from the analyses is that different strategy usage is required on problems which also involve the use of trial +A (according to our coding of sub-strategies), which is not among the effective sub-strategies for problems without autonomic changes. Analyses of students' behavior on problems with autonomic changes will form part of further studies, as well as a refinement of the definition of what makes a problem complex and difficult. We plan to adapt the Person, Task and Situation framework published by Beckmann and Goode (2017). The role of *ad hoc* control behavior was excluded from the analyses; further studies are required to ascertain the importance of the repetitive control behavior. Another limitation of the study could be the interpretation of the differences across age group clusters as indicators of development and not as a lack of stability of the model employed.

These results shed new light on and provide a new interpretation of previous analyses of complex problem solving in the MicroDYN approach. They also highlight the importance of explicit enhancement of problem-solving skills and problem-solving strategies as a tool for applying knowledge in a new context during school lessons.

## Ethics statement

Ethical approval was not required for this study based on national and institutional guidelines. The assessments which provided data for this study were integrated parts of the educational processes of the participating schools. The coding system for the online platform masked students' identity; the data cannot be connected to the students. The results from the no-stakes diagnostic assessments were disclosed only to the participating students (as immediate feedback) and to their teachers. Because of the anonymity and no-stakes testing design of the assessment process, it was not required or possible to request and obtain written informed parental consent from the participants.

## Author contributions

Both the authors, GM and BC, certify that they have participated sufficiently in the work to take responsibility for the content, including participation in the concept, design and analysis as well as the writing and final approval of the manuscript. Each author agrees to be accountable for all aspects of the work.

### Conflict of interest statement

The authors declare that the research was conducted in the absence of any commercial or financial relationships that could be construed as a potential conflict of interest.

## Footnotes

^1^With regard to terminology, please note that different terms are used for the subject at hand (e.g., complex problem solving, dynamic problem solving, interactive problem solving and creative problem solving). In this paper, we use the modifier “complex” (see Csapó and Funke, 2017; Dörner and Funke, 2017).
